# Diagnostic accuracy and prognostic applications of CYFRA 21-1 in head and neck cancer: A systematic review and meta-analysis

**DOI:** 10.1371/journal.pone.0216561

**Published:** 2019-05-09

**Authors:** Lihui Liu, Wenji Xie, Pei Xue, Zixuan Wei, Xiao Liang, Nianyong Chen

**Affiliations:** 1 Department of Head & Neck Oncology, Cancer Center and State Key Laboratory of Biotherapy, West China Hospital, Sichuan University, Chengdu, Sichuan, China; 2 Sleep Medicine Center, Department of Respiratory and Critical Care Medicine, Translational Neuroscience Center, State Key Laboratory, West China Hospital, Sichuan University, Chengdu, Sichuan, China; 3 Department of Neurosurgery of Huashan Hospital, Fundan University, Shanghai, China; Queen Mary University of London, UNITED KINGDOM

## Abstract

Cytokeratin fraction 21–1 (CYFRA 21–1) has been widely studied as an important biomarker in non-small cell lung cancer for both diagnosis and prognosis. Many studies have also assessed the clinical applications of CYFRA 21–1 in head and neck cancer, but the diagnostic and prognostic values of CYFRA 21–1 are not yet fully established. This pooled analysis aims at evaluating the diagnostic accuracy and prognostic applications of CYFRA 21–1 in patients with head and neck cancer. A systematic retrieval of literatures was conducted without time or language restrictions by searching PubMed, EMBASE, Web of Science, Cochrane library and China National Knowledge Infrastructure. Twenty studies were eligible for systematic review, of which 14 conformed for diagnostic analysis and 7 for prognostic analysis. The pooled sensitivity and specificity of CYFRA 21–1 analysis were 0.53 (95% CI: 0.39–0.67) and 0.97 (95% CI: 0.93–0.99), respectively. A high level of CYFRA 21–1 was significantly correlated with shorter overall survival (HR 1.33, 95% CI: 1.13–1.56) and disease-free survival (HR 1.48; 95%CI: 1.10–1.97). Current evidence indicates that the level of CYFRA 21–1 in the serum could be used as an indicator for monitoring tumor status and evaluating its curative effects.

## Introduction

Head and neck cancer (HNC) broadly include carcinomas derived from the mucosal epithelium of the head and neck region[1]. The risk factors for the development of oral, oropharyngeal, hypopharyngeal, and laryngeal cancers include tobacco exposure and alcohol dependence, and infection with Epstein-Barr virus is associated with cancers that develop in the nasopharynx[2–4]. Approximately 60% of newly diagnosed patients with HNC present with advanced disease (stage III/IV)[5]. Moreover, nasopharyngeal carcinoma (NPC) is one of the most aggressive HNC, which arises from the nasopharynx epithelium and has a unique geographical distribution[6–8]. The incidence of NPC has reached 27.2/100,000 among males and 11.3/100,000 among females in the South of China, which accounts for more than 60% of newly cases diagnosed cases worldwide[9–13]. Until now, the major treatments for most patients with HNC include radiotherapy alone or in combination with chemotherapy, but local recurrence still remains the main reason for treatment failure in this group of patients. About 50% of patients have local recurrence after primary tumor resection, and despite active treatment, 25% of them develop distant metastases[14–18]. Therefore, early diagnosis and precise prognosis of patients with HNC are critical and urgently required.

A serum marker, called cytokeratin fraction 21–1 (CYFRA 21–1), has shown a promising diagnostic value for HNC as well as other malignancies[19, 20]. CYFRA 21–1 is a cytoplasmic protein fragment of cytokeratin 19 (CK-19) which is found in various epithelial malignancies, such as lung cancer[21, 22], gastrointestinal cancer[23–25] and cervical cancer[26, 27]. This soluble debris can be released into the blood after tumor cell death, thus exhibiting a close relationship with tumor cell necrosis. Its level could be a proper indicator of necrosis degree. It has been reported that CYFRA 21–1 is a potential tumor marker for the diagnosis and prognosis of non-small cell lung cancer (NSCLC) and squamous carcinoma of the head and neck. Other studies have confirmed that the serum CYFRA 21–1 level before treatment is related to poor prognosis in patients with non-metastatic HNC[19].

With the ambiguity that exists for the clinicopathological role of CYFRA 21–1 in HNC, here, this study conducted a meta-analysis of published literatures on this topic to investigate the diagnostic and prognostic value of serum CYFRA 21–1 in HNC. In addition, we conducted a subgroup to assess the clinical value of CYFRA 21–1 in patients with NPC.

## Materials and methods

### Search strategy

A systematical retrieval of literature was conducted without time restriction. By using the keywords “cytokeratin fraction 21–1” and “CYFRA 21–1” variably combined with one of the following terms: “head and neck cancer”, “head and neck squamous cell carcinoma”,”oropharynx”,”nasopharynx”,”laryngopharynx” and”hypopharynx”, we searched all the articles in PubMed, EMBASE, Web of Science, Cochrane library and China National Knowledge Infrastructure databases. The final search was conducted on 7th July 2018 with an additional search in Google Scholar to supplement all the publications we needed.

### Inclusion and exclusion criteria

To make our analysis repeatable and reliable, studies were selected according to certain inclusion and exclusion criteria. The inclusion criteria were: 1) original research published in a peer-reviewed journal; 2) patients must be histologically confirmed with HNC and without other malignancies; 3) studies investigating the association between CYFRA 21–1 and survival outcomes or clinical characteristics of HNC patients; 4) sufficient data to extract or calculate hazard ratio (HR), odds ratio (OR) and 95% confidence intervals (95% CIs); and 5) available data for calculating sensitivity and specificity. The exclusion criteria were: 1) studies based on overlapping patients; 2) reviews, case reports, meta-analysis, communications, or letters were excluded; 3) studies with a poor sample size, here defined as ≤ 20 patients enrolled; and 4) outcome is not clear. Two authors (Liu and Xie) reviewed the selected studies independently.

### Data extraction and quality assessment

All data were extracted independently by two independent investigators (Xue and Wei), and any disagreement was settled through discussion. Data retrieved from the studies included the first author, year of publication, area distribution of patients, sample size of the study, follow-up duration of the study, information of clinicopathological characteristics (gender, age, stage, lymph node infiltration, distant metastasis, and recurrence), HR, prognostic value (OS and DFS), Kaplan-Meier curves for survival, 95% CI for survival, CYFRA 21–1 level, etc. If the HR and its 95% CI for OS or DFS were not reported, we extracted the data from the original study to calculate the HR. The methodological quality of the included studies for diagnostic analysis was evaluated by using the Quality Assessment of Diagnostic Accuracy Studies Tool and Newcastle-Ottawa Scale was applied to assess the quality of study for prognostic analysis.

### Statistics analysis

Fisher’s exact (I^2^) and Chi-square (Q) tests were used to assess the heterogeneity across included studies. The fixed-effects model was used to calculate the estimates if there was no significant heterogeneity observed. Otherwise, the random-effects model was applied. Besides, threshold effect analysis was also performed using Meta-DiSc software to explore the sources of heterogeneity. Diagnostic accuracy variables, including sensitivity, specificity, positive likelihood ratio (PLR), negative likelihood ratio (NLR), diagnostic odds ratio (DOR), and area under curve (AUC) of the summary receiver operating characteristic curve (SROC) were pooled and analyzed using Stata 15.1. To evaluate the prognostic value of CYFRA 21–1, HRs with 95% CIs were used to calculate the pooled HR. We evaluated potential publication bias of the pooled data using Deek’s funnel plot and Egger’s test, and *p*<0.05 was considered of significant publication bias. Moreover, we conducted a sensitivity analysis on prognostic value to explore whether the results were stable and reliable. All statistical analyses were performed using Stata 15.1 and Meta-DiSc.

## Results

### Literature search and study characteristics

**Fig 1:**
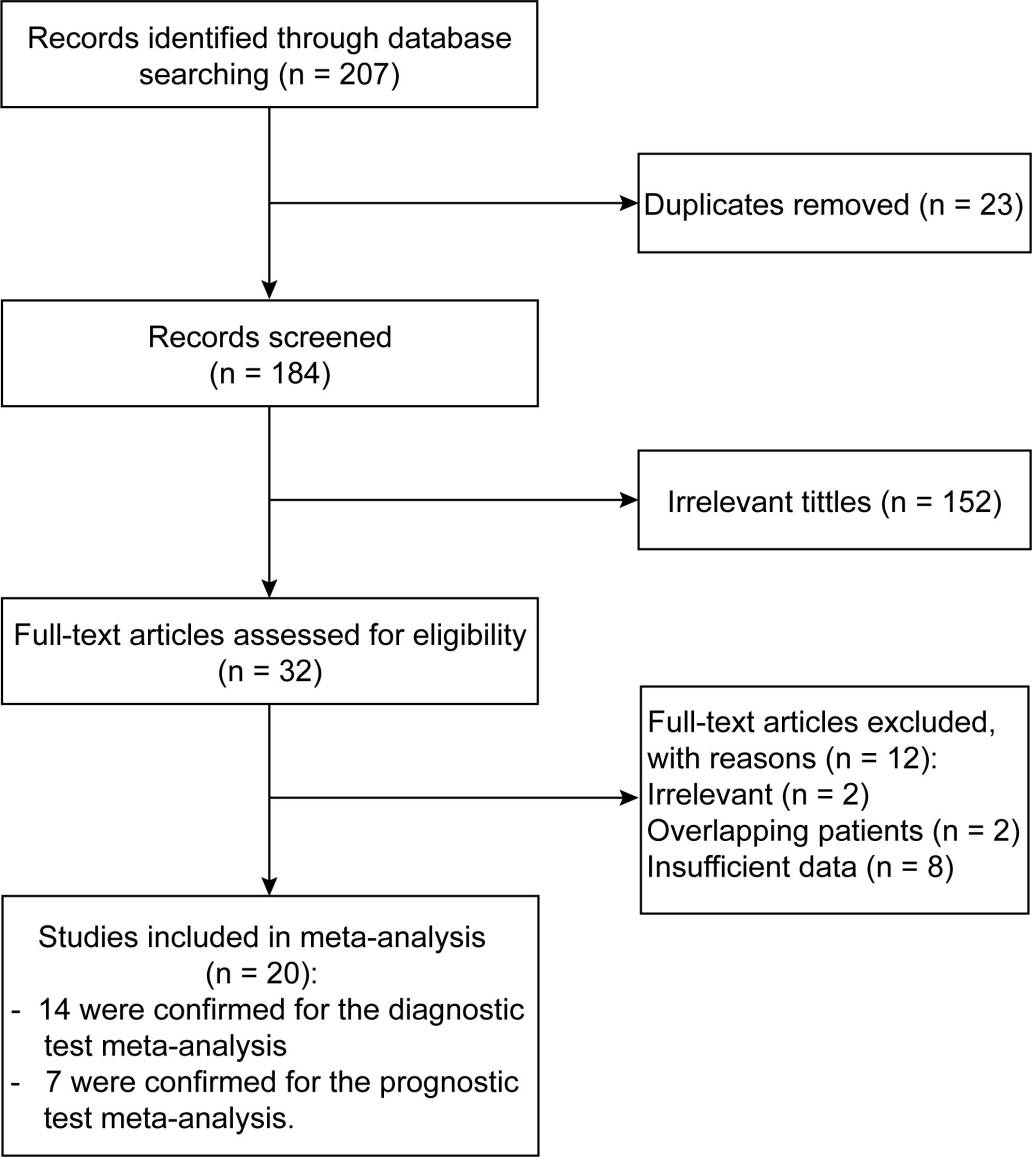
Flow chart of literature search and selection schema. The whole process follows PRISMA guidelines.

As shown in Fig 1, 207 studies were retrieved initially for systematic review, of which 187 were excluded because they were repeated or did not the inclusion criteria after reviewing the title and the abstract. Finally, 20 studies published between 1998 to 2018 that met our criteria were included after reading the full text[28–47]. The main characteristics of the 20 included studies for diagnostic analysis and prognostic analyses are listed in Table 1 and Table 2, respectively. The total number of patients included in all the studies was 3,796, and the mean sample size was 190 (ranged from 48 to 449 in each individual study). Of all the included studies, 14 studies were conducted for detecting the diagnostic value of CYFRA21-1 in HNC, while 7 studies were focused on investigating the relationship between CYFRA 21–1 and prognosis in patients with HNC. In all the 20 studies, electrochemiluminescent immunoassay (ECLIA) was the most frequently used method for detecting CYFRA 21–1 (in 8/20 studies), followed by immunoradiometric assay (IRMA) used in 7/20 studies, and enzyme-linked immunosorbent assay (ELISA) used in 5/20 studies.

**Table 1:** Basic characteristics of the retrieved studies for diagnostic analysis.

First author	Year	Area	Disease	Sample size		Method	Cut-off value (ng/mL)	QUADAS
				HNC	non-HNC			
Wollenberg[28]	1996	Germany	HNSCC	163	94	ELISA	2.90	10
Yen[29]	1998	China	HNSCC	168	77	IRMA	2.48	9
Lee[30]	2001	China	NPC	80	77	IRMA	2.48	10
Tai[31]	2002	China	NPC	60	43	IRMA	2.50	11
Ayude[32]	2003	Spain	HNSCC	40	101	ECLIA	1.70	10
Deng[33]	2003	China	HNSCC	142	118	ECLIA	3.30	10
Inal[34]	2004	Turkey	HNSCC	28	20	IRMA	NA	9
Céruse*[35]	2005	France	HNSCC	300	71	IRMA	1.00	10
Huang[36]	2005	China	NPC	82	50	ELISA	3.36	8
Eleftheriadou[37]	2006	Greece	HNSCC	79	80	ECLIA	3.30	10
Kandiloros[38]	2006	Greece	HNSCC	136	125	ECLIA	3.30	10
Zhong[39]	2007	China	OSCC	100	56	ELISA	0.65	12
Malhotra[40]	2016	China	OSCC	50	50	ECLIA	3.00	12
Song[41]	2016	India	NPC	274	175	ELISA	3.09	12

Abbreviations: QUADAS, Quality Assessment of Diagnostic Accuracy Studies; HNC, head and neck cancer; HNSCC, head and neck squamous cell carcinoma; NPC, nasopharyngeal carcinoma; LSCC, laryngeal squamous cell carcinoma; OSCC, oral squamous cell carcinoma; ELISA, enzyme-linked immunosorbent assay; IRMA, immunoradiometric assay; ECLIA, electrochemiluminescent immunoassay; NA, not available.

*Céruse, 2005: this research studied both the diagnostic and prognostic value of CYFRA 21–1.

**Table 2:** Basic characteristics of the retrieved studies for prognostic analysis.

First author	Year	Disease	Sample size	Median of follow-up (months)	Tumor stage	Sample site/time	Mean age (years)	Method	NOS
Brigette[42]	2004	NPC	160	29	I–IV	PB/pre-TM	46	IRMA	8
Céruse[35]	2005	HNSCC	300	33	I–IV	PB/pre-TM	48	IRMA	8
Zhu[43]	2010	NPC	61	45	I–IV	PB/pre-/post-TM	46	ELISA	7
Lei[44]	2014	NPC	89	35	II–IV	PB/post-TM	NA	ECLIA	7
Wei[45]	2014	NPC	332	48	I–IV	PB/pre-TM	42	IRMA	9
Hsu[46]	2015	OSCC	130	19	I–IV	PB/pre-TM	52	ECLIA	6
Jolanta[47]	2018	HNSCC	185	40	I–IV	PB/pre-/post-TM	59	ECLIA	9

Abbreviations: NOS, Newcastle-Ottawa Scale; HNSCC, head and neck squamous cell carcinoma; NPC, nasopharyngeal carcinoma; OSCC, oral squamous cell carcinoma; TM, treatment; ELISA, enzyme-linked immunosorbent assay; IRMA, immunoradiometric assay; ECLIA, electrochemiluminescent immunoassay; NA, not available.

### Meta-analysis of diagnostic accuracy

**Fig 2:**
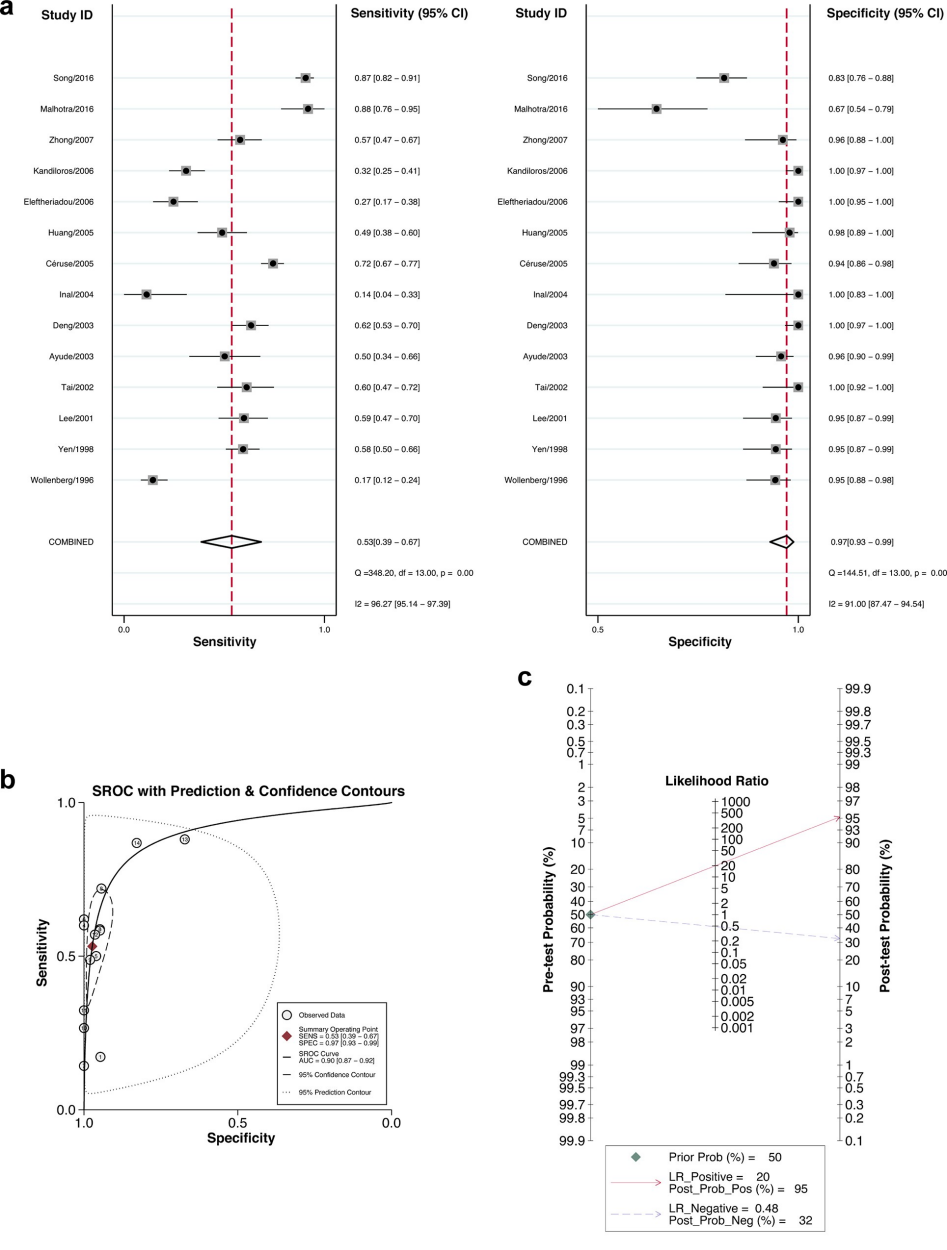
Diagnostic accuracy of CYFRA 21–1 in HNC. Forest plots of sensitivity and specificity of CYFRA 21–1 for the diagnosis of HNC. Studies are labeled by the names of the first author and the published year (a). The regression SROC curve and the AUC of the SROC curve indicate the overall diagnostic accuracy (b). Fagan plot analysis to evaluate the clinical application of CYFRA 21–1 in the diagnosis of HNC (c).

The 14 eligible studies were pooled for the meta-analysis of diagnostic test (Table 1). As significant heterogeneity among the 14 studies was observed (I^2^ = 96.27%, *p*<0.001 for Sensitivity and I^2^ = 91.00%, *p*<0.001 for Specificity), we chose the random-effects model to synthesize the data. The sensitivity and specificity of pooled studies were 0.53 (95% CI: 0.39–0.67) and 0.97 (95% CI: 0.93–0.99), respectively (Fig 2A). The PLR and NLR were 19.8 (95% CI: 9.1–43.1) and 0.48 (95% CI: 0.32–0.64), respectively. The pooled DOR was 41 (19–87) and the AUC of the SROC curve was 0.90, indicating a high overall accuracy (Fig 2B). We performed Fagan plot analysis to further assess the clinical value of CYFRA 21–1 for the diagnosis of HNC, which revealed that CYFRA 21–1 might serve as a good indicator of HNC (Fig 2C).

Moreover, we conducted a subgroup analysis within the studies on NPC (I^2^ = 95.20%, *p*<0.001 for Sensitivity and I^2^ = 83.90%, *p*<0.001 for Specificity) to evaluate the diagnostic accuracy of CYFRA 21–1 in NPC. In this case, the pooled sensitivity and specificity were 0.66 (95% CI: 0.48–0.80) and 0.95 (95% CI: 0.87–0.98), respectively. The PLR and NLR were 14.3 (95% CI: 5.9–35.1) and 0.36 (95% CI: 0.23–0.56), respectively. The DOR was 40 (19–85) and the AUC of the SROC curve was 0.92. All the results of diagnostic meta-analysis are summarized in Table 3.

**Table 3:** Summary of subgroup analysis for diagnostic value.

	Sensitivity (95% CI)	Specificity (95% CI)	PLR (95% CI)(95% CI)	NLR (95% CI)(95% CI)	DOR (95% CI)(95% CI)	AUC (95% CI)(95% CI)
**HNC (except NPC)(except NPC)**	0.53 (0.40–0.66)	0.98 (0.96–0.99)	32.4 (14.1–74.2)	0.47 (0.36–0.63)	68 (33–140)	0.91 (0.89–0.93)
**NPC**	0.66 (0.48–0.80)	0.95 (0.87–0.98)	14.3 (5.9–35.1)	0.36 (0.23–0.56)	40 (19–85)	0.92 (0.89–0.94)
**IRMA**	0.53 (0.35–0.70)	0.97 (0.91–0.99)	16.8 (6.8–41.3)	0.48 (0.33–0.71)	35 (14–87)	0.94 (0.91–0.95)
**ECLIA**	0.54 (0.32–0.74)	0.99 (0.84–1.00)	79.2 (3.8–1638.8)	0.47 (0.29–0.75)	170 (10–2804)	0.86 (0.83–0.89)
**ELISA**	0.53 (0.25–0.79)	0.93 (0.86–0.97)	7.7 (4.8–12.2)	0.50 (0.27–0.92)	15 (7–33)	0.91 (0.88–0.93)

Abbreviations: PLR, positive likelihood ratio; NLR, negative likelihood ratio; DOR, diagnostic odds ratio; AUC, area under the curve; 95% CI: 95% confidence interval; HNC, head and neck cancer; NPC, nasopharyngeal carcinoma. ELISA, enzyme-linked immunosorbent assay; IRMA, immunoradiometric assay; ECLIA, electrochemiluminescent immunoassay.

To further look into the differences between the three different methods (IRMA, ECLIA, and ELISA), we performed a subgroup analysis of assays for detecting CYFRA 21–1 in the serum. According to our results, there was no significant difference observed in the detected sensitivity or specificity (Table 3).

### Meta-analysis of prognostic value

**Fig 3:**
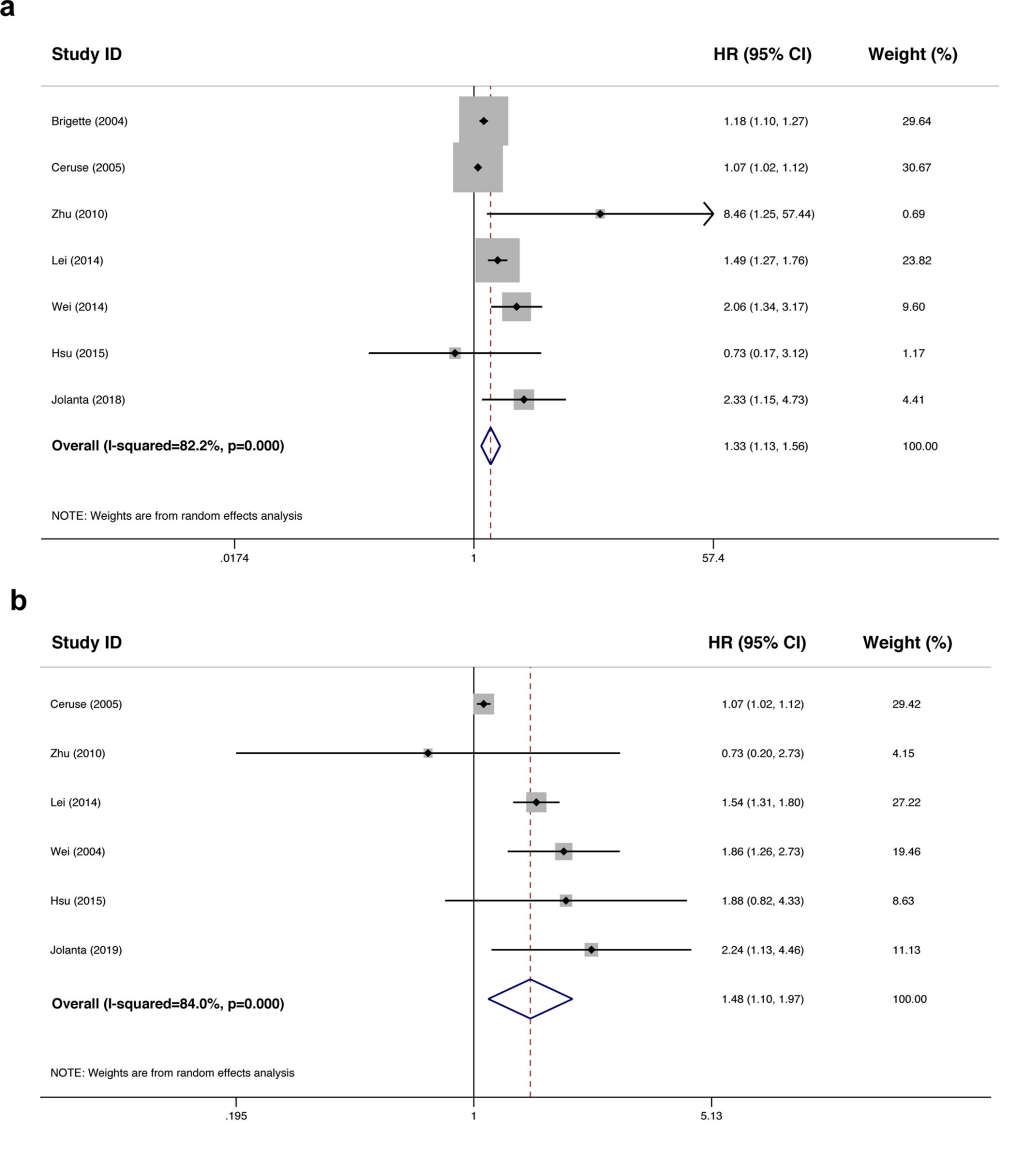
Forest plots showing the prognostic value of CYFRA 21–1 in patients with HNC patients. Meta-analysis estimating CYFRA 21–1 overexpression with OS (a) and DFS (b) using random-effects model.

In total, 7 eligible studies with available survival data and Kaplan-Meier survival curves were included for the meta-analysis of prognostic value and all the data were extracted directly from raw data without calculating or processing. Of these studies, 7 assessed OS and 6 evaluated DFS. With relatively obvious heterogeneity (I^2^ = 82.2%, *p*<0.001), a random-effects model was chosen for our analysis. The pooled HR was 1.33 (95% CI: 1.13–1.56; Fig 3A) and a significant difference was found for OS between the CYFRA 21-1-high level and -low level patients with HNC. For the DFS, 6 studies with significant heterogeneity (I^2^ = 84.0%, *p*<0.001) were analyzed using a random-effects model and a significant overall HR was found (the pooled HR was 1.48, 95% CI: 1.10–1.97; Fig 3B).

In addition, we conducted a subgroup analysis of the correlation between CYFRA 21–1 and prognosis of patients with NPC (I^2^ = 81.2%, *p* = 0.001). The resulting forest plots are showed in Table 4. The pooled HR for OS was 1.50 (95%CI: 1.14–1.97), indicating that high level of CYFRA 21–1 was closely related to a poor OS in our included studies. The parameters for DFS were calculated using a fixed-effects model according to the heterogeneity test (I^2^ = 4.1%, *p* = 0.353), and the pooled HR for DFS was 1.57 (95% CI: 1.33–1.84), suggesting that the tumor progression or recurrence rate in patients with NPC showing high level of CYFRA 21–1 was 1.57 times of that in patients with NPC showing low level of CYFRA 21. Taken together, patients with HNC showing high CYFRA 21–1 level tended to have a relatively poor prognosis. All the results of prognostic meta-analysis are summarized in Table 4.

**Table 4:** Summary of subgroup analysis for prognostic value.

Study	HR (95% CI) for OS	HR (95% CI) for DFS	*p* value
**HNC**			
Brigette 2004	1.18 (1.10–1.27)	NA	
Céruse 2005	1.07 (1.01–1.11)	1.07 (1.02–1.12)	
Zhu 2010	8.46 (1.25–57.44)	0.73 (0.19–2.73)	
Lei 2014	1.49 (1.27–1.75)	1.54 (1.31–1.80)	
Wei 2014	2.06 (1.34–3.17)	1.86 (1.26–2.73)	
Hsu 2015	0.73 (0.17–3.12)	1.88 (0.82–4.33)	
Jolanta 2018	2.33 (1.15–4.73)	2.24 (1.13–4.46)	
pooled	1.33 (1,13–1.56)	1.48 (1.10–1.97)	
Egger’s test			0.056
**NPC**			
Brigette 2004	1.18 (1.10–1.27)	NA	
Zhu 2010	8.46 (1.25–57.44)	0.73 (0.19–2.73)	
Lei 2014	1.49 (1.27–1.75)	1.54 (1.31–1.80)	
Wei 2014	2.06 (1.34–3.17)	1.86 (1.26–2.73)	
pooled	1.50 (1.14–1.97)	1.57 (1.33–1.84)	
Egger’s test			0.060

Abbreviations: HR, hazard ratio; 95% CI: 95% confidence interval; OS, overall, survival; DFS, disease-free survival; NA, not available; HNC, head and neck cancer; NPC, nasopharyngeal carcinoma.

Moreover, we performed a subgroup analysis of prognostic value based on the cut-off value of CYFRA21-1 in patients with HNC. We found no significant relationship between OS and the cut-off value of 3.00 ng/mL, while DFS was significantly correlated with the cut-off value of 3.00 ng/mL. When cut-off value of CYFRA21-1 was greater than 3.00 ng/mL, the pooled HR for DFS was 1.59 (95% CI: 1.37–1.84), but a cut-off value less than 3.00 ng/mL showed no significant correlation between DFS and CYFRA 21–1 (S1 Fig).

### Heterogeneity and meta-regression analysis

**Fig 4:**
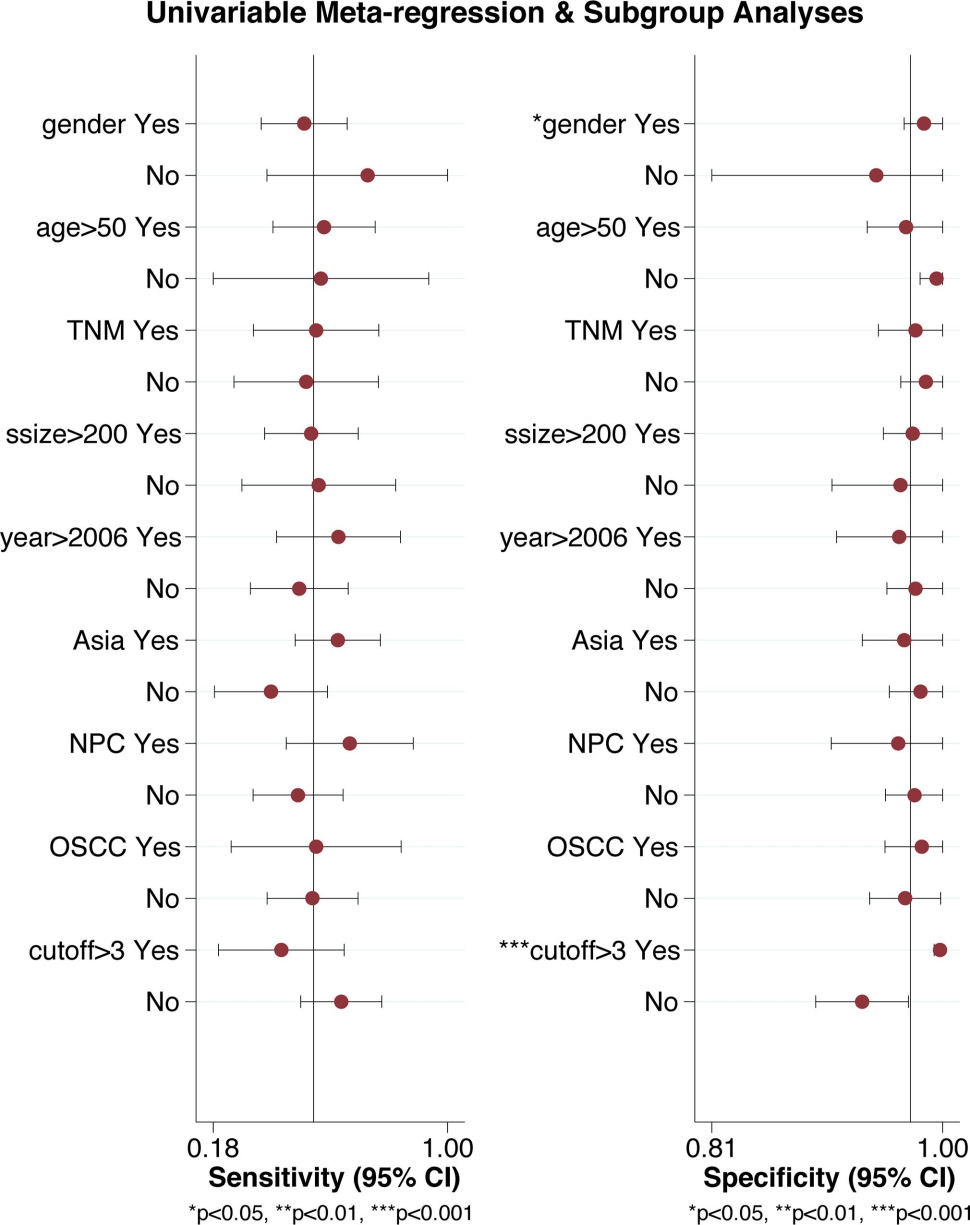
Meta-regression analysis of heterogeneity. Meta-regression analysis on covariates, including gender (male : female > 0.6), age (> 50), TNM stage (T1+T2 > T3+T4), sample size (> 200), published date (> 2006), study region (Asia or not), NPC (NPC or not), OSCC (OSCC or not), and cut-off value (> 3.00 ng/mL).

There was obvious heterogeneity found among included studies especially in the cut-off value of CYFRA 21–1 ranging from 1.00 ng/mL to 3.36 ng/mL (Table 1), and for this reason, we calculated Spearman’s correlation coefficient and *p* value to assess the threshold effect in our diagnostic meta-analysis by using Meta-Disc software. The overall Spearman’s correlation coefficient appeared to be 0.475 and the *p* value was 0.086 (>0.05), indicating that there was no significant threshold effect. To find the sources of heterogeneity, we conducted meta-regression analysis on covariates, including gender (male : female > 0.6), age (> 50), TNM stage (T1+T2 > T3+T4), sample size (> 200), published date (> 2006), study region (Asia or not), NPC (NPC or not), OSCC (OSCC or not), and cut-off value (> 3.00 ng/mL). However, there was no significant correlation between these covariates and heterogeneity in sensitivity. We found that a cut-off value more than 3.00 ng/mL and a male/female ratio greater than 0.6 were sources of heterogeneity in specificity (Fig 4).

### Sensitivity analysis and publication bias

**Fig 5:**
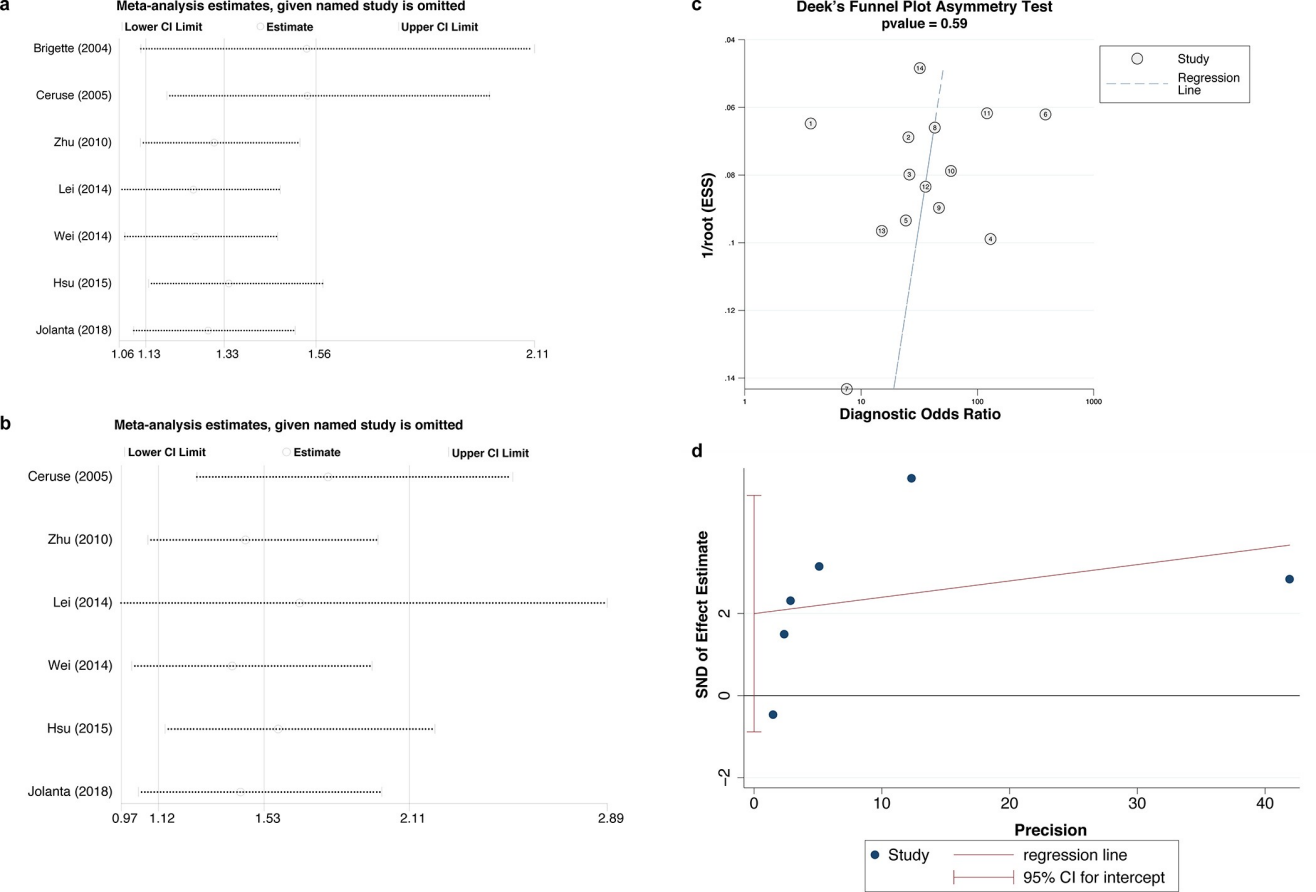
Sensitivity analysis and publication bias of meta-analysis. Sensitivity analysis for the pooled prognostic value for OS (a) and DFS (b). Deek’s funnel plot asymmetry test shows a low likelihood of publication bias in diagnostic meta-analysis (c). Egger’s test shows no significant publication bias in prognostic meta-analysis (d).

As a limited number of studies were included in the analysis for prognostic value, and the heterogeneity for the prognostic value was relatively obvious, we conducted sensitivity analysis for the pooled prognostic value to evaluate whether our results would be severely affected by some high-weight studies. The pooled results showed no significant changes after omitting any one of the included studies for both OS (Fig 5A) and DFS (Fig 5B). Besides, all the results were consistent with our previous data for prognosis, confirming that high level of CYFRA 21–1 was associated with poor prognosis.

The Deek’s funnel plot asymmetry test was used to assess the likelihood of publication bias for the diagnostic value of CYFRA 21–1. The slope coefficient was associated with a *p* value of 0.59, indicating a symmetry in the eligible data and a low likelihood of publication bias (Fig 5C). At the same time, we used Egger’s test to evaluate the potential publication bias of the final set of studies for prognostic meta-analysis (Fig 5D). Finally, we found no significant publication bias in all the variables analyzed in this study (Table 4, *p*>0.05).

## Discussion

HNC is a common malignant disease with relatively high morbidity and mortality[1, 5]. Although the advances in radiotherapy technology have greatly improved the therapeutic effects on patients with HNC, local recurrence still remains the main reason for treatment failure in this group of patients and more than 25% of them finally develop distant metastases[14, 15, 48]. Therefore, early diagnosis and precise prognosis of patients with HNC are very important and urgently needed. Our study confirms that CYFRA 21–1 can serve as a potential indicator for diagnosis of HNC as well as an effective biomarker for predicting the prognosis of patients with HNC.

In this study, we firstly made a comprehensive meta-analysis of the diagnostic value of CYFRA 21–1 in HNC. SROC curve was applied to recapitulate the overall test performance of CYFRA 21–1, and our results demonstrated that CYFRA 21–1 detection in patients with HNC has limited sensitivity but very high specificity (0.97). The AUC of 0.90 and DOR of 41 reported in our study prove the diagnostic accuracy of CYFRA 21–1 in HNC. To more intuitively illustrate the clinical application of CYFRA 21–1 in the diagnosis of HNC, we also conducted Fagan plot analysis. Taking together, detection of CYFRA 21–1 level would be of great help for the diagnosis of patients suspected with HNC.

Furthermore, we conducted a meta-analysis of eligible studies to assess the prognostic value of CYFRA 21–1, and provided strong evidence that patients with HNC with a relatively high level of CYFRA 21–1 in the serum tended to have poor prognosis. In the 7 studies we included in analyzing the prognostic value, all patients received conventional radical radiotherapy, and those with advanced stage (stage IIB, III, or IV) tumors also received concomitant or adjuvant chemotherapy. There were no significant differences in treatment modalities between tumor subsites. For OS, we found that the risk of death in CYFRA 21-1-high level patients was 1.33 times higher than that in CYFRA 21-1-low level patients (pooled HR: 1.33, 95% CI: 1.13–1.56). As for DFS, the risk of tumor progression and relative death in CYFRA 21-1-high level patients was 1.48 times of that in CYFRA 21-1-low level patients (pooled HR was 1.48, 95% CI: 1.10–1.97). Taken together, detection of CYFRA 21–1 level in patients with HNC could predict prognosis and help screen patients with a relatively high recurrence or tumor progression rate.

Moreover, since NPC is one of the most aggressive types of HNC, which arises from the nasopharynx epithelium, with a high incidence in China, we conducted a subgroup analysis of both diagnostic and prognostic values of CYFRA 21–1 for patients with NPC. Our data showed that the pooled sensitivity and specificity for diagnosis of NPC were 0.66 and 0.96, respectively. The pooled DOR was 42 and the AUC of the SROC curve was 0.92, indicating high accuracy. The pooled HR for OS was 1.50 while the pooled HR for DFS was 1.57. Thus, our results suggested that CYFRA 21–1 might also serve as an indicator for the diagnosis and prognosis of patients with NPC.

CYFRA 21–1 is a fragment of CK-19, which is widely distributed in epithelial cells, and a high-level of serum CYFRA 21–1 indicates degradation of tumor-transformed epithelial cells, leading to the release of these CK-19 fragments into the blood[40, 49]. Of note, CYFRA 21–1 has been widely studied as an important biomarker of NSCLC in both diagnosis and prognosis[50–54]. A retrospective analysis of 1,990 patients with NSCLC previously revealed that the risk of death in CYFRA 21-1-high level patients is 1.64 times higher than that in CYFRA 21-1-low level patients (pooled HR for OS: 1.64, 95% CI: 1.46–1.84)[54]. Besides, a recent study has also found that CYFRA 21–1, rather than carcinoembryonic antigen and neuron-specific enolase, is more important for metastasis occurrence in patients with lung cancer[55]. Interestingly, these results were in accordance with our findings on HNC, confirming that CYFRA 21–1 plays an important role in both tumorigenesis and metastasis in squamous cell carcinoma.

Despite the great potential of CYFRA 21–1 in both diagnosis and prognosis of patients with HNC, there are still many challenges. First, there are many methods for detecting CYFRA 21–1, including IRMA, ECLIA and ELISA. However, due to the limited number of studies, it was not yet possible for us to compare and conclude which method is the best. In this study, we utilized existing data and made a general comparison between three detection methods. There were no significant differences in sensitivity or specificity between the methods; however, we still need more systematic and comprehensive researches to draw a more accurate conclusion. Second, most of the studies we retrieved detected CYFRA 21–1 level merely at baseline (pre-treatment, 54%) and did not follow up the level of CYFRA 21–1 during and after treatment. Therefore, a large amount of useful information may have been omitted, making it difficult for us to analyze the dynamic changes of CYFRA 21–1 throughout the disease. Finally, there was obvious heterogeneity, especially in the cut-off value of CYFRA 21–1, among the included studies. According to our clinical knowledge and experience, we conducted subgroup analyses of NPC and the detection methods to reduce the heterogeneity, and there was no significant threshold effect observed in our analysis. Moreover, we performed meta-regression analysis on covariates, including gender, age, TNM stage, sample size, published date, study region, disease, and cut-off value, but found no significant correlation between these covariates and heterogeneity. Taking into account these results, large-scale multicenter studies using the same detection method and standard as well as inclusion and exclusion criteria should be carried out to reduce heterogeneity and a standard detection method as well as a cut-off value for CYFRA 21–1 should be set.

## Conclusions

Our study comprehensively demonstrated the diagnostic and prognostic value of CYFRA 21–1 in patients with HNC. Current evidence suggests that CYFRA 21–1 is an extremely specific indicator of HNC. Despite its limitation of sensitivity, a combination with other indicators would greatly increase its diagnostic accuracy for HNC. A high level of CYFRA 21–1 in patients with HNC can predict poor prognosis. The serum level of CYFRA 21–1 could be served as an indicator for monitoring tumor status and evaluating its curative effects.

## Supporting information

S1 Fig Subgroup analysis for prognostic value based on whether the cut-off value of CYFRA 21–1 was greater than 3.00 ng/mL. (TIF)

S1 File PRISMA checklist to this study. (DOC)
