# Supplementary figures and images for: Diagnostic accuracy and prognostic applications of CYFRA 21-1 in head and neck cancer: A systematic review and meta-analysis
Source: PLoS One. 2019 May 9;14(5):e0216561. doi: 10.1371/journal.pone.0216561 (PMC6508679; doi:10.1371/journal.pone.0216561)

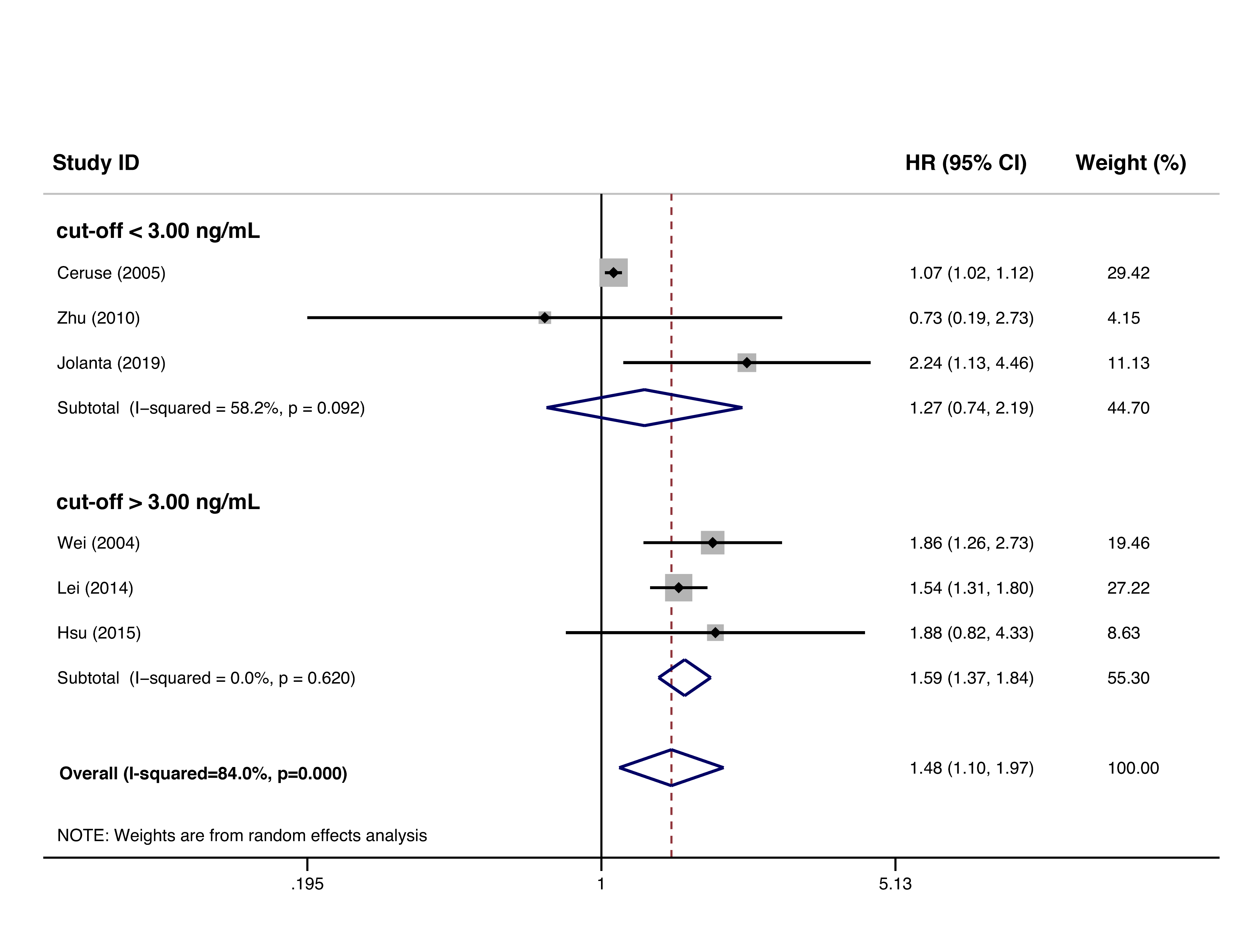

Supplement: S1 Fig — (TIF) [file pone.0216561.s001.tif]
